# A physical model for low-frequency electromagnetic induction in the near field based on direct interaction between transmitter and receiver electrons

**DOI:** 10.1098/rspa.2016.0338

**Published:** 2016-07

**Authors:** Ray T. Smith, Fred P. M. Jjunju, Iain S. Young, Stephen Taylor, Simon Maher

**Affiliations:** 1 Department of Electrical Engineering and Electronics, University of Liverpool, Liverpool L69 3GJ, UK; 2 Institute of Integrative Biology, University of Liverpool, Liverpool L69 3BX, UK

**Keywords:** coils, electromagnetic induction, propagation, wireless power transfer, solenoids, transformers

## Abstract

A physical model of electromagnetic induction is developed which relates directly the forces between electrons in the transmitter and receiver windings of concentric coaxial finite coils in the near-field region. By applying the principle of superposition, the contributions from accelerating electrons in successive current loops are summed, allowing the peak-induced voltage in the receiver to be accurately predicted. Results show good agreement between theory and experiment for various receivers of different radii up to five times that of the transmitter. The limitations of the linear theory of electromagnetic induction are discussed in terms of the non-uniform current distribution caused by the skin effect. In particular, the explanation in terms of electromagnetic energy and Poynting’s theorem is contrasted with a more direct explanation based on variable filament induction across the conductor cross section. As the direct physical model developed herein deals only with forces between discrete current elements, it can be readily adapted to suit different coil geometries and is widely applicable in various fields of research such as near-field communications, antenna design, wireless power transfer, sensor applications and beyond.

## 1. Introduction

Near-field electromagnetic (EM) interactions are used in various application areas such as magnetic induction (MI) communications [1], MI tomography [2,3] and wireless power transfer [4]. They are being increasingly employed in wireless underground sensor networks [5,6] for applications such as environmental monitoring (in soil [7] and water [8]), landslide inspection [9] and underground pipeline surveillance [10]. Traditional wireless sensor approaches are inhibited by the complex propagation media encountered (e.g. soil, rock, water). However, by taking advantage of near-field, low-frequency magnetic fields, difficulties associated with propagation delay, fading and multipath propagation are not as prominent. The term near field relates to the non-radiative propagation over short distances of magnetic or electric fields owing to inductive or capacitive coupling, respectively. By contrast, the far-field refers to radiative EM fields at large distances from the source, which has received extensive coverage [11–14].

There have been several valuable research initiatives modelling EM fields in the near-field region which usually involve exact representations and/or computationally intensive routines [15–23], which according to Mikki & Antar [24], ‘cannot lead to significant insights on general questions, such as the nature of electromagnetic radiation or the inner structure of the antenna near field’. Nevertheless, magnetic near-field modelling is an important task, for example, when designing the complex circuits to determine compliance with EMC standards [25].

In this paper, we develop a method for the case of a multi-turn finite transmitter and receiver coil pair of circular geometry arranged concentrically. The basis for this method, which has been adapted to calculate the induced emf in the receiver at some distance from the source, is the Weber force formula that can be considered as a modification of Coulomb’s law for charges in relative motion [26–32]. This force relates directly to the force between moving charges in terms of their displacement, relative radial velocity and relative radial acceleration in a discrete system.

Defining the limits of the near-field region is an ambiguous task as it depends on the geometry and excitation of the transmitter in question. Mikki & Antar rightly stress, in their detailed and comprehensive review of antenna theory in the near field, the ‘need of a sustained, comprehensive, and rigorous treatment for the topic of near fields, a treatment that takes into account the peculiar nature of the electromagnetic behaviour at this zone [24]’. It is generally accepted that ‘the near field’ includes, at the very least, the surrounding space up to a distance of one wavelength and may well extend further. We also present the preliminary results of how induced voltage varies with both distance and frequency based on particle–particle interactions in this zone.

First, we explore the theoretical basis for low-frequency EM induction. In doing so, we develop a direct action model that relates directly to the current distribution in the finite transmitter and receiver coils. The model is corroborated with experimental measurements by calculating the receiver response at increasing distances from the source. Finally, we discuss the advantages and limitations of the model and provide suggestions for further research.

## 2. Theory

**Figure 1.**
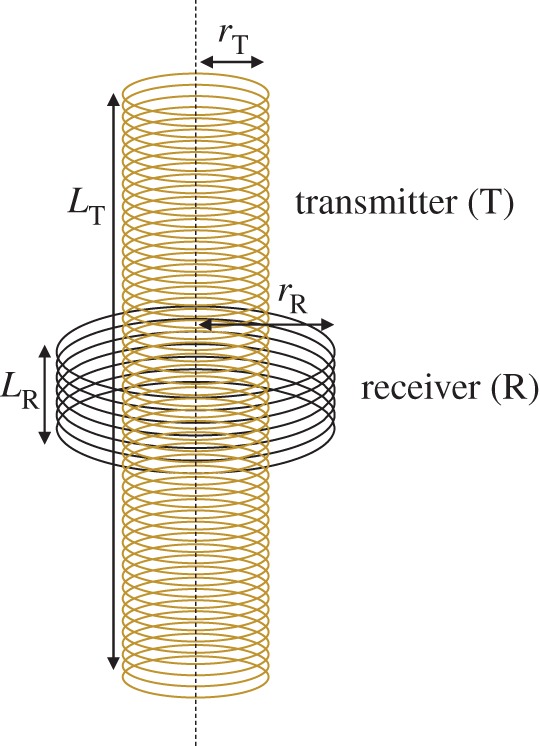
Configuration of the coaxial air-core finite coils. The transmitter (T) of length, *L*_T_, radius, *r*_T_ with *N*_T_ closely wound turns is situated inside the receiver (R) of length, *L*_R_, radius, *r*_R_ and *N*_R_ closely wound turns. (Online version in colour.)

The case of EM induction under consideration is sometimes referred to as transformer induction. The arrangement consists of coaxial coils arranged concentrically with the transmitter (T) given by the inner coil and the receiver (R) by the outer as illustrated in figure 1.

### (a) Faraday’s law of induction

Faraday’s law relates the induced emf, *e*, in a closed circuit to the rate of change of magnetic flux through that circuit. This is generally given by

2.1 $$e=-\frac{d\emptyset }{dt},$$

where ∅ is the magnetic flux (n.b. equation (2.1) is strictly only valid for wire of infinitely small cross section). For the arrangement of figure 1, the transmitter coil is supplied with an alternating current, $I=I_{0}\sin\omega t$, where *I*_0_ is the peak current and *ω* is the radial frequency given by, *ω* = 2*πf*. The magnetic flux through the receiver follows the current such that ∅=∅_0_sin 2*πft*, and the induced emf in the receiver is given by

2.2 $$e_{R}=-\frac{d\emptyset }{dt}=-\emptyset_{0}2\pi f\;\cos(2\pi ft).$$

For the case of an infinite multi-turn coil, the magnetic flux density in the central region is given by *B*=*μ*_0_*nI*_0_, where *μ*_0_ is the permeability of free space and *n* is the winding density (=*N*/*L*). The peak magnetic flux per turn linking the transmitter and receiver is given by the product of the flux density and the cross-sectional area of a single turn (*Bπr*^2^_T_). Assuming that the receiver is wound closely on to the transmitter such that *r*_R_−*r*_T_≅0, then the peak emf induced in the receiver is given by

2.3 $${\left(e_{R}\right)}_{0}=-\frac{2\pi^{2}r_{T}^{2}n_{T}N_{R}I_{0}f}{\varepsilon_{0}c^{2}},$$

where *ε*_0_=permittivity of free space, *c*=speed of light and *n*_T_ is the transmitter turn density.

### (b) Neumann’s mutual inductance formula

A more general method for calculating induced emf between closed circuits can be obtained from Neumann’s formula. Assuming the magnetic flux density is proportional to the current (Biot–Savart law) and expressing the flux in terms of the vector potential (***A***), then for closed loops *T* and *R* with elements d***l***_T_, d***l***_R_ at a distance *r* apart

$$\emptyset_{R}=\oint A_{T}\cdot dl_{R},$$

where

$$A_{T}=\frac{\mu_{0}I_{0}}{4\pi }\oint \frac{dl_{T}}{r}$$

So that

$$\emptyset_{R}=\frac{\mu_{0}I_{T}}{4\pi }\oint \left(\oint \frac{dl_{T}}{r}\right)\cdot dl_{R}$$

Because ∅_R_=*M*_RT_*I*_T_, where *M*_RT_=*M*_TR_ which is the mutual inductance of the two loops, then the Neumann formula is given by

2.4 $$M_{\mathrm{RT}}=\frac{\mu_{0}}{4\pi }\oint \oint \left(\frac{1}{r}\right)dl_{T}\cdot dl_{R}.$$

The mutual inductance between the two closed circuits is a geometrical quantity relating to the size, shape and relative positions of the two loops and is independent of whichever circuit is acting as the transmitter or receiver. Re-writing Faraday’s law by taking account of the mutual inductance (*M*), alternating transmitter current *I* and the associated changing magnetic flux, the induced emf in the receiver is given by

$$e_{R}=-\frac{d\emptyset }{dt}=-M\frac{dI}{dt}.$$

Assuming, as before, that the receiver is wound closely on to the transmitter such that *r*_R_−*r*_T_≅0. Then, noting that, *M*=*μ*_0_*πr*^2^_T_(*N*_T_/*l*_T_) and $I=I_{0}\;\sin\omega t$, with the number of receiver turns acting as a multiplying factor, the peak-induced emf in the receiver is given as

$${\left(e_{R}\right)}_{0}=-\frac{2\pi^{2}r_{T}^{2}n_{T}N_{R}I_{0}f}{\varepsilon_{0}c^{2}}$$

which is the same as (2.3).

### (c) Grover’s solution when *r*_T_≠*r*_R_

A specific solution for concentric coaxial coils of different radii is given in reference [33] by

$$M=0.004\pi^{2}r_{T}^{2}n_{T}N_{R}(B_{1}r_{1}-B_{2}r_{2}),$$

where $r_{1}=\sqrt{r_{R}^{2}+(1/4){\left(l_{T}+l_{R}\right)}^{2}}$ and $r_{2}=\sqrt{r_{R}^{2}+(1/4){\left(l_{T}-l_{R}\right)}^{2}}$

The functions *B*_1_ and *B*_2_ depend on the parameters, $p_{1}^{2}=r_{R}^{2}/r_{1}^{2}$, $p_{2}^{2}=r_{R}^{2}/r_{2}^{2}$, *α*=*r*_T_/*r*_R_ and can be obtained from tables in reference [33]. For example, using specific coil data in the present experiment, values for *M* were calculated as 9.22 mH (*r*_T_/*r*_R_=1) and 8.66 mH (*r*_T_/*r*_R_=0.78), giving an increase of approximately 6% when the receiver is assumed to be closely wound.

### (d) Vector potential

The vector potential outside a long solenoid is derived as, *A*=∅/2*πr*, where ∅ is the total magnetic flux inside the transmitter coil. The electric field outside the transmitter is then *E*=−∂*A*/∂*t*=−(1/2*πr*)(d∅/*dt*). Equating ∅=*LI*=*μ*_0_*nIπr*^2^_T_, where *L* is the inductance per unit length of an infinite multi-turn coil and *n* is the number of turns per unit length, then for a single loop of radius *r*_R_ encircling the transmitter

$$E=-\frac{1}{2}(\mu_{0}nr_{T}2\pi f)I_{0}\cos\omega t.$$

Integrating around a single loop and including a multiplying factor to account for *N*_R_ receiver turns

$${\left(e_{R}\right)}_{0}=\oint E.dl=2\pi r_{T}E=-\frac{2\pi^{2}r_{T}^{2}n_{T}N_{R}I_{0}f}{\varepsilon_{0}c^{2}}$$

which, again, is (2.3).

## 3. Direct action approach

**Figure 2.**
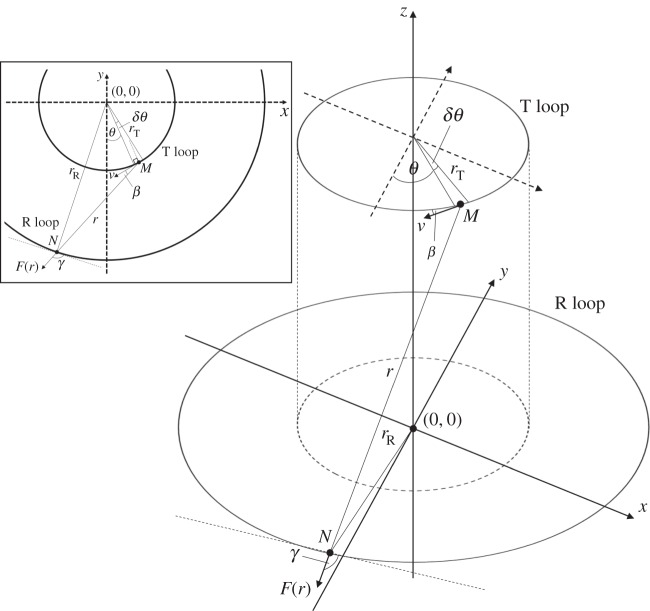
Geometry of single circular loops making up part of the transmitter and receiver coils. The inset shows the geometry projected onto a two-dimensional plane (*x*–*y*).

Consider two single circular transmitter (T) and receiver (R) loops. The transmitter loop is of radius *r*_T_ and is excited by an alternating current of a given frequency *f*, whereas the encircling receiver loop is of radius *r*_R_ across which an emf is induced (*r*_R_>*r*_T_) as illustrated in figure 2.

Using a Cartesian coordinate system, we define the centre of the receiver loop as the origin. In applying Weber’s force law to this case, the force is determined between a line element of charge (=*r*_T_*δθ*) in the transmitter located at point *M* and a unit charge located in the receiver at point *N*, where the distance between these points is given as *MN*=*r*. Using Weber’s force formula, adapted in terms of relative velocity [32], the force resolved along *r* is given as

3.1 $$F_{r}=\frac{q_{P}}{4\pi \varepsilon_{0}}\frac{\hat{\text{r}}}{r^{2}}\left[1+\frac{1}{c^{2}}\left(u^{2}-\frac{3}{2}u_{r}^{2}+r\frac{d^{2}r}{dt}\right)\right],$$

where *q*_*M*_=*n*′*Ar*_T_*δθe*, is an element of charge at *M*, *n*’ is the electron density, *A* is the area of wire cross section, *e* is electron charge, $\hat{\text{ r}}$ is a unit vector along *r*, *u*_*r*_ is relative velocity along *r* and *u* is the relative velocity between *M* and *N*. For this case, whereby there is no net flow of current in the receiver, the relative velocity between *M* and *N* is given by the electron drift velocity, *v*, at *M* in the transmitter loop. Hence, in (3.1), *u*^2^=*v*^2^. The relative velocity along *r* is given as $u_{r}=dr/dt=v\;\cos\beta =vb\;\sin\theta /r$. Both *u*^2^ and $u_{r}^{2}$ terms which appear in (3.1) involve *v*^2^ terms that can be ignored for small currents leaving only the acceleration term, *r*(d^2^*r*/d*t*)=*r*(d*u*_*r*_/d*t*) and *r* is determined by trigonometry, $r^{2}=r_{T}^{2}+r_{R}^{2}-2r_{T}r_{R}\;\cos\theta +z^{2}$. By differentiating *u*_*r*_ and noting that *v*=*r*_T_(d*θ*/d*t*), ignoring *v*^2^ terms, yields $r\;du_{r}/dt=r_{R}\;\sin\theta (dv/dt)$. Because *I*=*n*′*Ave*, then $\dot{v}=\dot{I}/n^{\prime }Ae$ and therefore

3.2 $$F_{r}=\frac{r_{T}r_{R}\;\sin\theta }{4\pi \varepsilon_{0}c^{2}r^{2}}\dot{I}.$$

Resolving along the tangent to the receiver loop, gives force per unit charge as, $E_{T}=F_{r}\;\cos\gamma$, where $\cos\gamma =-r_{T}\;\sin\theta /r$ and therefore,

3.3 $$E_{T}=-\frac{r_{T}^{2}r_{R}\dot{I}}{4\pi \varepsilon_{0}c^{2}}\frac{\sin^{2}\theta }{r^{3}}\delta \theta .$$

The induced emf in the receiver is given by integrating around the closed loop so that

3.4 $$e_{r}=\oint E.dl=2\pi r_{R}E_{T}=-2\pi \frac{r_{T}^{2}r_{R}^{2}\dot{I}}{4\pi \varepsilon_{0}c^{2}}\frac{\sin^{2}\theta }{r^{3}}\delta \theta .$$

By differentiating the transmitter current, we obtain peak-induced emf in a single receiver loop as

3.5 $${\left(e_{R}\right)}_{0}=\frac{\pi r_{T}^{2}r_{R}^{2}I_{0}}{\varepsilon_{0}c^{2}}f\int_{0}^{2\pi }\frac{\sin^{2}\theta }{r^{3}}d\theta .$$

To compute the induced emf in a finite multi-turn coil, the principle of superposition is applied to current contributions from each individual coil turn. The integrand from (3.5) is computed for a range of *z*-values from each turn. The *z*-values relate to the vertical distance between turns given in terms of the wire diameter, *d*. Then using standard numerical integration (trapezium rule at 5^°^ intervals) gives

3.6 $${\left(e_{R}\right)}_{0}=\frac{2\times \pi r_{T}^{2}r_{R}^{2}I_{0}f}{\varepsilon_{0}c^{2}}\sum \left[\begin{array}{ccc} e(-314d) & \cdots & e(335d)\\ \vdots & \ddots & \vdots \\ e(-333d) & \cdots & e(316d) \end{array}\right],$$

where $r^{2}=r_{T}^{2}+r_{R}^{2}-2r_{T}r_{R}\cos\theta +z^{2}$. The factor of 2 takes into account the contributions from both layers of the transmitter (i.e. transmitter coil is doubly wound). The matrix has 20 (*N*_R_) rows by 650 (*N*_T_/2) columns representing all of the individual turn contributions, where the *z*-value is equal to zero for the case when individual transmitter, and receiver coil turns are directly aligned. The summation in (3.6) is for all of the individual terms in the matrix. For example, the summation of the first row of the matrix, gives the induced voltage in the first receiver turn from all of the 650 individual transmitter turns (see appendix A).

## 4. Experimental

In order to verify the above-mentioned approach, the following experimental measurements were carried out. The experimental set-up consists of a finite coaxial inner transmitter coil and an outer receiver coil as depicted in figure 1. The transmitter (inner) coil of length, *L*_T_=0.5 m, consists of 1300 turns doubly wound with single core-enamelled copper wire of 0.7 mm diameter and winding *density*=2600 turns per metre on a former of radius, *r*_T_ approximately 0.0292 m. Three receiver coils were used each with the same turn density and number of turns (*N*_R_=20) but with different radii, *r*_R_ of 0.0375, 0.075 and 0.15 m.

**Figure 3.**
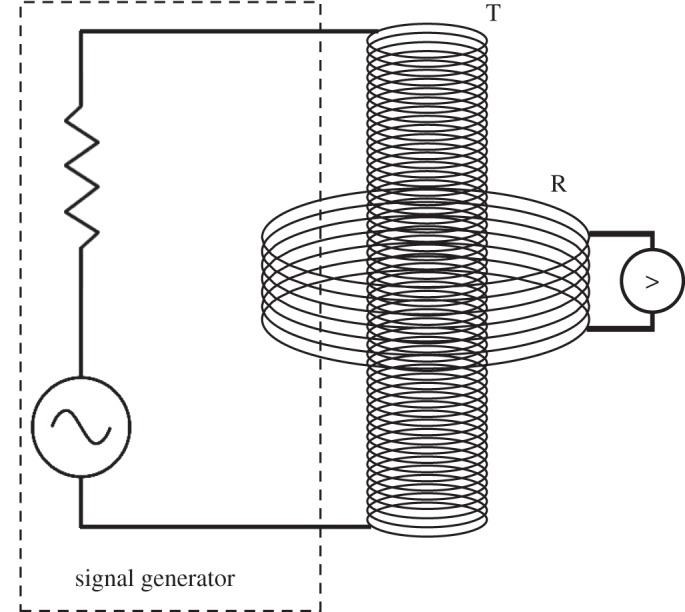
Circuit diagram of the experimental set-up for measuring the induced emf in the receiver coil.

The transmitter coil was connected to a digital signal generator (Lascells, UK) providing a sinusoidal transmitter current of 3 mA rms, measured by a Keithley 5.5 digit multimeter, across the 0–14 kHz frequency range. The receiver-induced voltage was measured simultaneously with a digital oscilloscope (Tektronix, USA). The basic circuit schematic is shown in figure 3. To improve the SNR, the receiver coil was screened from outside interferences around its circumference using mu metal shielding (fully heat treated, 0.35 mm thick, ASTM A753 Alloy 4, Magnetic Shields, UK). All calculations were computed using Matlab 2014a (MathWorks, USA).

## 5. Results

**Figure 4.**
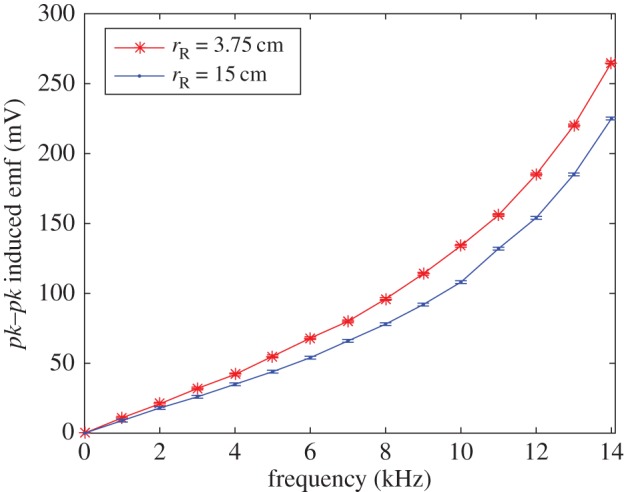
Induced emf (*pk*–*pk*, mV) response against frequency for receiver radii of 3.75 and 15 cm. (Online version in colour.)

**Figure 5.**
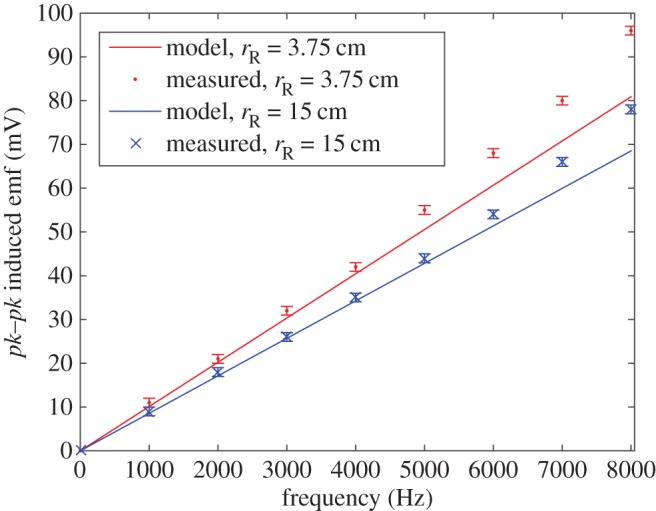
Induced emf (*pk*–*pk*, mV) response against frequency over the range 1–8 kHz compared with calculated data for receiver radii of 3.75 and 15 cm. (Online version in colour.)

**Figure 6.**
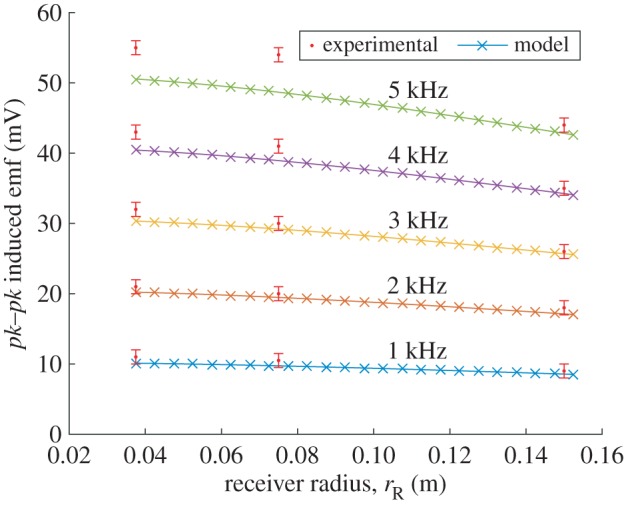
Calculated induced emf (*pk*–*pk*, mV) response against receiver radius for a range of frequencies supplemented with measurements for receiver radii of 3.75, 7 and 15 cm for *f*≤5 kHz. (Online version in colour.)

The experimental results are summarized in figures 4–6. Figure 4 shows the induced receiver emf (peak-to-peak) plotted against frequency for two different receiver coil radii of 3.75 and 15 cm. The smaller receiver coil is less than approximately 1 cm from the transmitter coil outer surface, whereas the larger receiver coil is approximately 12 cm from the transmitter coil with a diameter approximately five times greater than the transmitter coil. Both coils follow the same trend with the induced emf larger for the smaller radii coil (i.e. closer to the transmitter) than the larger for each measurement. Initially, the trend of induced emf versus frequency follows a linear response. This is seen clearly in figure 5 which also includes the modelled data, calculated using (3.6) with equivalent model parameters. In figure 6, the calculated model trend against receiver radii is compared with measured results for the three different experimental receiver coil radii (*r*_R_=0.0375, 0.075, 0.15 m) at various frequencies.

## 6. Discussion

The advantage of the Weber-based formulation is that it can readily accommodate different receiver and transmitter radii. The comparison between experiment and theory is shown in figures 5 and 6 where the modelled data are within limits of experimental error, in the linear regime (*f*<*approx*. 5 kHz). According to the model, the emf reduces with increasing receiver radii owing to reduced interelectron forces as the coupling between the coils reduces with distance.

The nonlinearity associated with induced emf at higher frequencies is evident in figure 4. Above approximately 5 kHz, the response departs from linear behaviour. From (3.5), it can be seen that the direct action model depends linearly on the frequency as is the case with Faraday’s law as given in (2.3). For Faraday’s law, the induced emf depends on the rate of change of current, hence the linear dependence on *f*. Similarly, for the direct action approach, the relative electron accelerations also depend linearly on the frequency. To the best of authors’ knowledge, there does not appear to be any satisfactory theory that can deal with the case of variable frequency EM induction. Feynman [34] discusses attempts to modify Maxwell’s equations, all of which encounter difficulties associated with the assumption of point charges, the self-action of a charge on itself (radiation reaction) and the part played by EM mass as opposed to mechanical mass.

For the direct action model, the assumption is made that current is uniformly distributed over the cross section of the wire and that the principle of superposition applies to successive coil sections. With increasing frequency, any linear induction model will break down as the current distribution becomes non-uniform giving rise to the well-known skin and proximity effects. The phenomenon that has become known as the skin effect was discovered by Maxwell who hypothesized non-uniform current distribution [35]. The high-frequency resistance can be given as a dc resistance of an equivalent ‘skin’ with a certain depth of penetration. The proximity effect relates to the current interferences between individual adjacent loops, because the geometrical form of the field is not constant but changes with frequency. This presents a considerable challenge for any model of EM induction. However, the direct action approach has the intrinsic advantage that it can accommodate higher-order acceleration terms. At higher frequencies, the theory might be adapted to model thin tubes of current rather than making the assumption of uniform current density.

Various concepts and solutions have been developed to determine the skin effect for a range of conditions [36–42]. A more direct and physical explanation of the skin effect is in terms of the greater inductance (electron inertia) of filaments near the centre of the conductor compared with those at the surface. That is, current reversals at the central filaments experience higher resistance/reactance compared with those at the surface. Therefore, as the frequency increases, the current becomes more restricted to the outer regions of the conductor. A possible physical basis for the skin effect has been suggested in terms of electromagnetic mass (*M*_*e*_). Cullwick [43] has suggested that the effective conduction electrons charge is not the charge of all the available conduction electrons and that the current is carried by a small number of electrons travelling with high velocity. Following this reasoning, inductance can be regarded as the analogue of electromagnetic mass. Grover [33], in contrast, describes the skin effect in the following terms: ‘Electromagnetic energy enters the surface of the wire and is more and more attenuated and retarded in phase as the centre is approached. At very high frequencies, the attenuation is so great that the current amplitude becomes inappreciable after the wave has penetrated into the wire only a fraction of a millimetre’. This is essentially the explanation based on Poynting’s theorem according to which energy supplied to a conductor carrying current does not flow through the wire but through the surrounding EM field [44].

As there is at present no satisfactory general, nonlinear theory of EM induction, it is useful to fit the induced emf-frequency data by some form of empirical law. There is a linear variation up to approximately 5 kHz consistent with Weber’s law. Above approximately 5 kHz, with skin effect becoming progressively more significant, higher-order frequency terms are involved. Using the Matlab curve fitting tool, the following expressions are obtained for the values above 5 kHz (figure 4),

6.1 $${\left(e_{R}\right)}_{pk-pk}\langle r_{R}=0.0375\rangle =2.3f^{2}-22.4f+127.5$$

and

6.2 $${\left(e_{R}\right)}_{pk-pk}\langle r_{R}=0.15\rangle =2.2f^{2}-23.1f+124,$$

where the induced emf is peak-to-peak given in mV and frequency is given in kHz. A quadratic response is obtained with the coefficient of determination (*R*^2^) calculated as 0.9983 and 0.9986 for equations (6.1) and (6.2), respectively. Such empirical fits, for given geometries, might prove useful as a basis for comparison with mutual inductance over a range of frequencies.

Finally, it is worth commenting on why any nonlinear theory of induction proves difficult. A conduction electron subject to an alternating force is set into forced vibration in which it is subject to both restoring and damping forces. As the forcing frequency increases and electron flow becomes restricted to the outer regions of the conductor, then the same current through a reduced area will cause increased electron drift velocity and therefore increased vibration amplitude. This then gives rise to a nonlinear restoring force (i.e. not too dissimilar to a spring which may become ‘harder’ or ‘softer’ in a mechanical system). The consequence is that harmonic motion at small amplitudes can become an harmonic at large amplitudes and so give rise to higher-order frequency terms which are then required to describe the variation of secondary coil voltage.

## 7. Conclusions

For the coaxial coil arrangement studied the direct action approach shows good agreement with experimental measurements for predicting the induced emf in a receiver coil at various distances from the transmitter in the near field (up to five times the diameter of the transmitter coil). The model is of interest beyond the arrangement studied herein as it could well be adapted to suit other coil geometries. The model takes into account the radius of each coil, applied frequency, amplitude of the excitation current and contributions from individual coil turns as given in (3.6).

The linearity between induced emf and frequency is shown to hold up to frequencies of approximately 5 kHz. Above this, the progressive restriction of current to the outer regions of the conductor (skin effect) gives rise to a nonlinear dependence of induced voltage with frequency. The data were found to conform to a quadratic dependence on frequency as given in (6.1) and (6.2). In regard to the skin effect, the standard field-centred explanation concludes that it involves a flow of EM energy sideways into a conductor according to Poynting’s theorem. The model developed in this study suggests that an alternative explanation related to the variation of electron inertia/inductance across the conductor. Recently, there has been renewed interest in hydrodynamic analogies of electron flow in specific materials with some evidence that electron viscosity plays an important role in determining electrical resistance [45]. In connection to this, it is interesting to note that there is also a hydrodynamic analogy to electrical skin depth associated with acoustic streaming in an air-filled tube in which a low-frequency pulsating flow is superimposed on an existing steady flow [46]. The particle velocity is shown to reach a maximum value at a distance from the tube wall given by, $d_{w}\approx \sqrt{\upsilon /\pi f}$, where *υ* is the kinematic viscosity. This is contrasted with electrical skin depth, $\delta =1/\sqrt{\pi f\mu_{0}\sigma }$. Because *μ*_0_ is constant and *ρ*=1/*σ*, then $\delta \approx \sqrt{\rho /\pi f}$ hence providing the analogy with electron viscosity and electrical resistance.

Future work will involve developing this model for other cases including specific applications of interest such as MI imaging as well as exploring the possibility of extending the model to include higher-order frequency terms. This approach is of interest beyond that studied here as it provides an alternative and possibly more efficient means of modelling EM induction in the near field that could be useful in allied fields such as near-field communications, radiofrequency identification and EM compatibility. The accuracy of the model prediction over an appreciable distance from the transmitter means that the arrangement might be adapted as a reference standard for calibration of field strength meters for receiving loop antennas. Furthermore, this method may have implications for studying the influence of near-field EM interactions with biological bodies. Because the Weber force formulation describes moving charges and these do not necessarily have to be electrons in a copper wire, the theory might well be extended to charged particles in motion [47–51] or ionic species in biomedical systems [52–54], in particular providing insights into the effects of EM induction on specific biological processes.

## Figures and Tables

**Figure 7.**
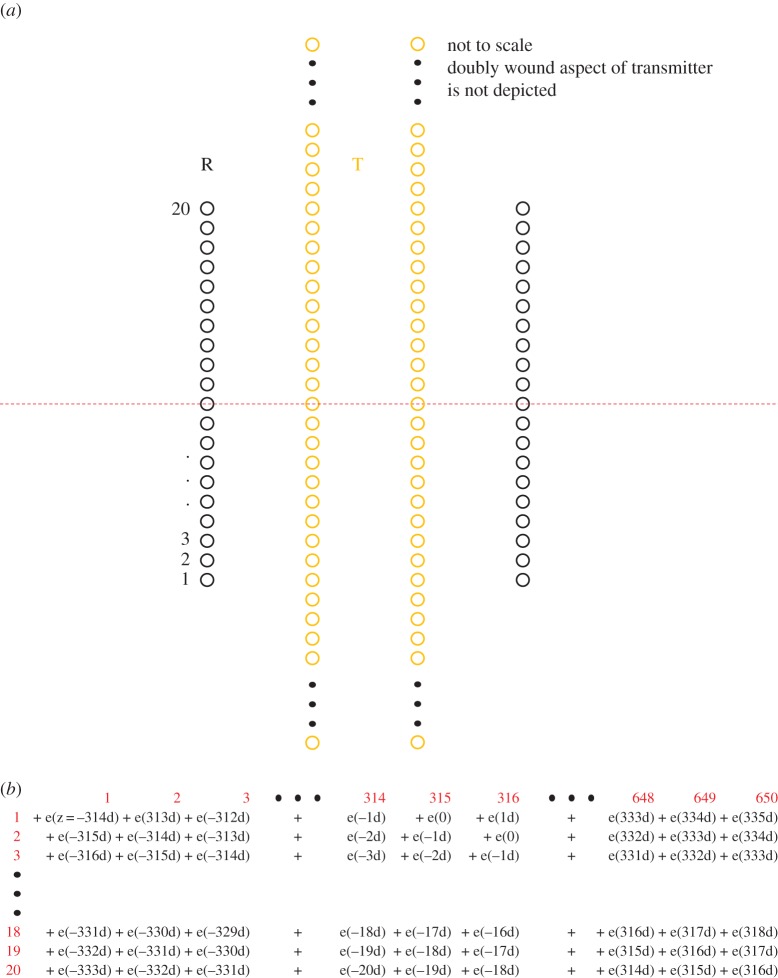
(*a*) Sketch shows the positions of individual turns in the transmitter–receiver coil arrangement. (*b*) An expansion of the matrix from equation (3.6) to illustrate the summation procedure. (Online version in colour.)

## Appendix A

Figure 7 shows individual transmitter–receiver turns as used in the summation of equation (3.6).
